# Supplementary material for: An Automated Image Analysis Pipeline Enables Genetic Studies of Shoot and Root Morphology in Carrot (Daucus carota L.)
Source: Front Plant Sci. 2018 Nov 27;9:1703. doi: 10.3389/fpls.2018.01703 (PMC6277879; doi:10.3389/fpls.2018.01703)
Supplement: Supplementary file 1 [file Image_1.pdf]

frontiers

Supplementary Material:

An automated, high-throughput image analysis pipeline enables genetic studies of shoot and root morphology in carrot (Daucus carota L.)

1 SUPPLEMENTARY FIGURES

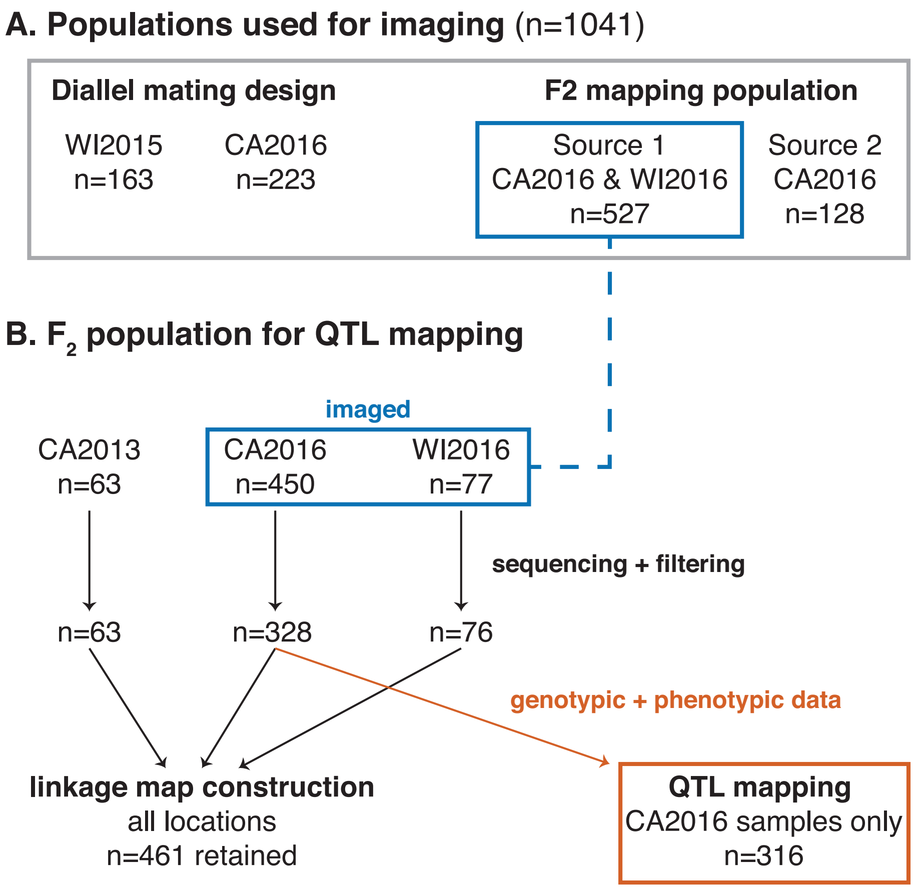

Figure S1. Diagram detailing the source populations and number of individuals used for imaging, genotyping, and QTL mapping.

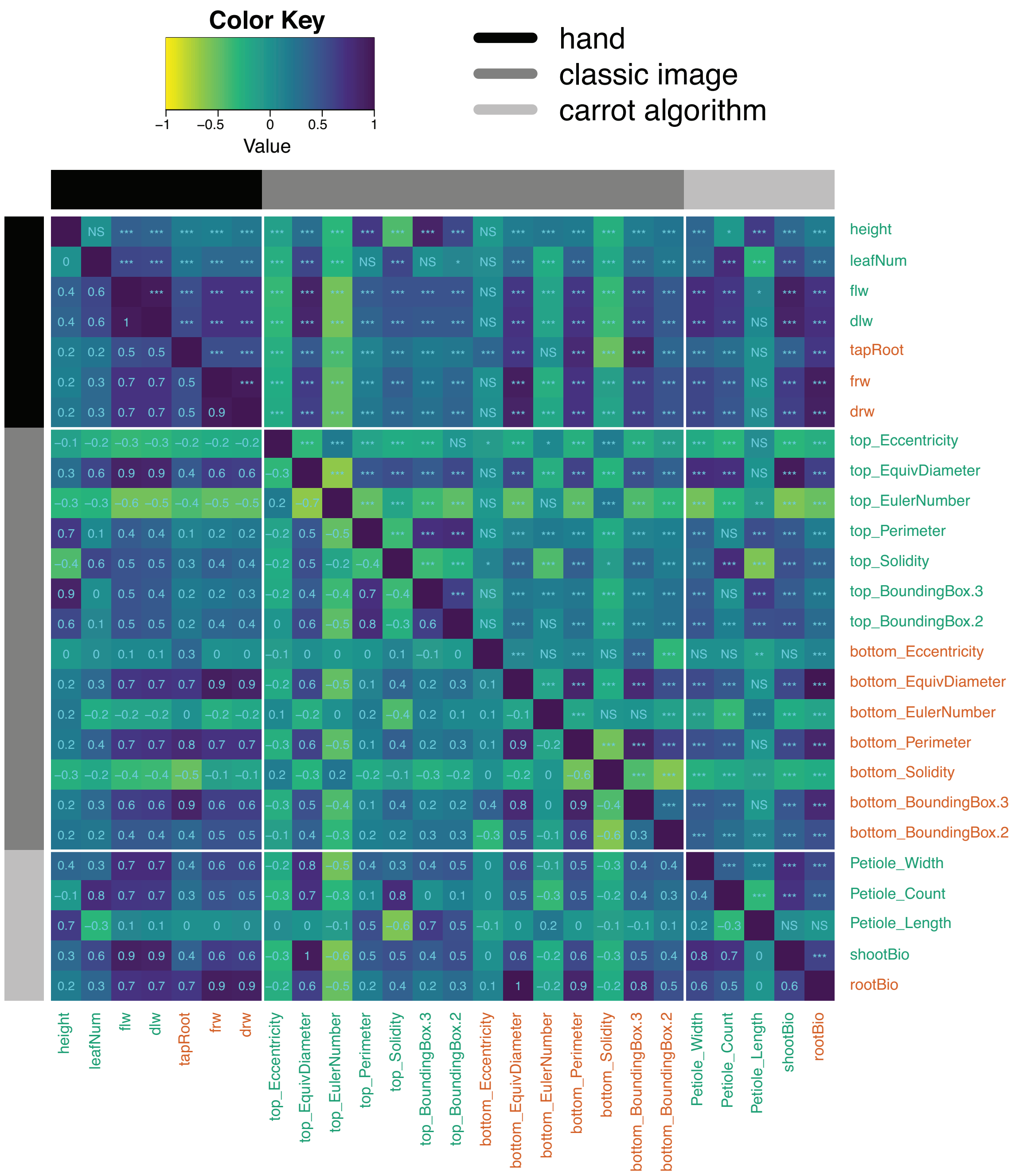

Figure S2. Pearson's correlations among hand measurements (black bar), classic image measurements (dark gray bar), and measurements predicted from the algorithm (light gray bar). The lower diagonal displays correlation values and the upper diagonal displays significance (***P≤0.001, **P≤0.01, *P≤0.05).

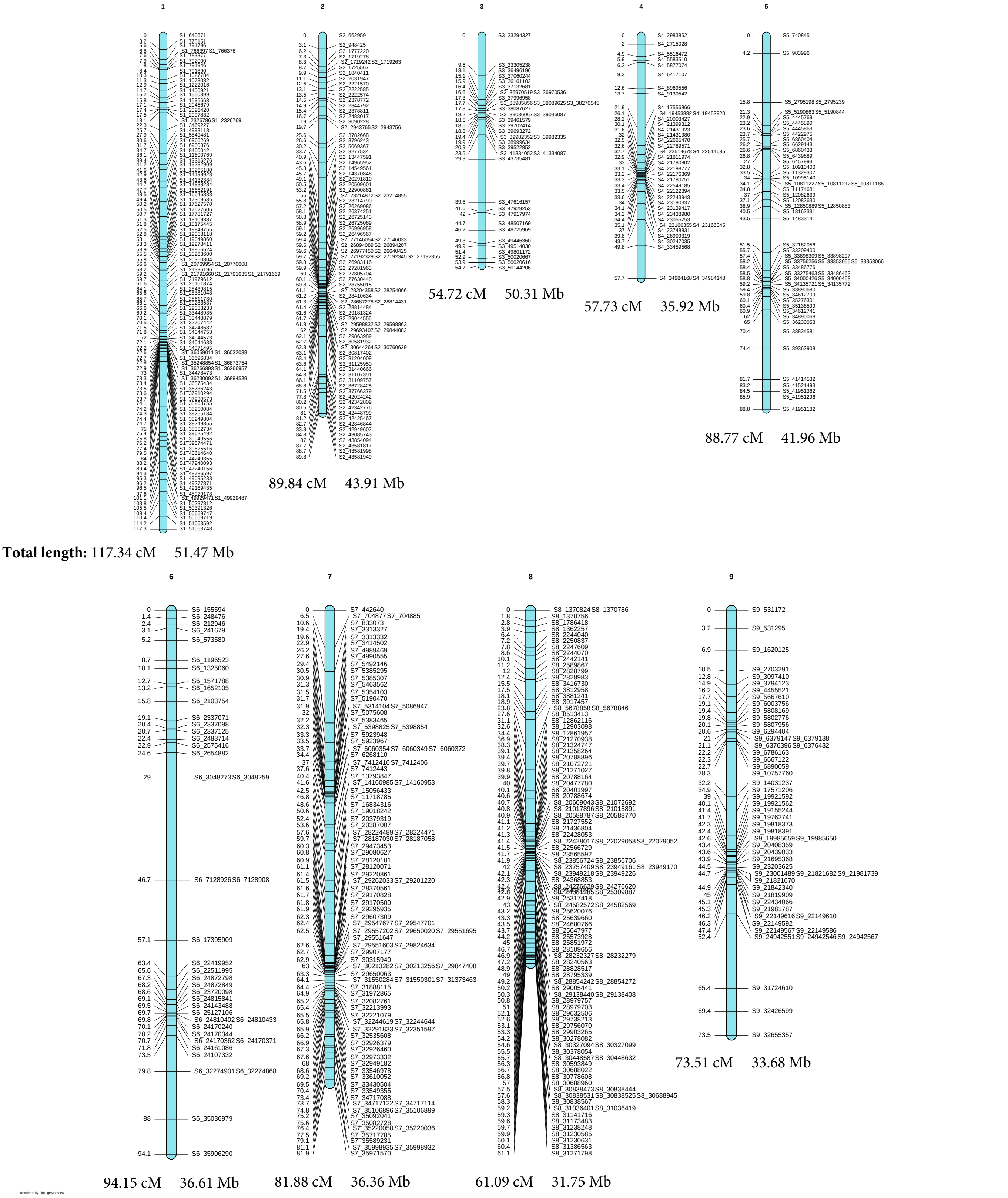

Figure S3. Linkage map for the L8708 x Z020 F_2 mapping population. Total length is provided in centimorgans (cM) and megabases (Mb) below each chromosome. This figure was generated using the LinkageMapView package in R 3.3.2.
